# A Review of Machine Learning for Near-Infrared Spectroscopy

**DOI:** 10.3390/s22249764

**Published:** 2022-12-13

**Authors:** Wenwen Zhang, Liyanaarachchi Chamara Kasun, Qi Jie Wang, Yuanjin Zheng, Zhiping Lin

**Affiliations:** 1 School of Electrical and Electronic Engnineering, Nanyang Technological University, Singapore 639798, Singapore; 2 School of Physical and Mathematical Sciences, Nanyang Technological University, Singapore 637371, Singapore

**Keywords:** machine learning, near-infrared spectroscopy, light absorption, non-invasive measurement, deep architectures

## Abstract

The analysis of infrared spectroscopy of substances is a non-invasive measurement technique that can be used in analytics. Although the main objective of this study is to provide a review of machine learning (ML) algorithms that have been reported for analyzing near-infrared (NIR) spectroscopy from traditional machine learning methods to deep network architectures, we also provide different NIR measurement modes, instruments, signal preprocessing methods, etc. Firstly, four different measurement modes available in NIR are reviewed, different types of NIR instruments are compared, and a summary of NIR data analysis methods is provided. Secondly, the public NIR spectroscopy datasets are briefly discussed, with links provided. Thirdly, the widely used data preprocessing and feature selection algorithms that have been reported for NIR spectroscopy are presented. Then, the majority of the traditional machine learning methods and deep network architectures that are commonly employed are covered. Finally, we conclude that developing the integration of a variety of machine learning algorithms in an efficient and lightweight manner is a significant future research direction.

## 1. Introduction

Infrared (IR) is an electromagnetic radiation that is divided into three categories based on their wavelengths: (1) near infrared (NIR) is defined as wavelengths between 0.78 and 2.5 μm; (2) mid infrared (MIR) is defined as wavelengths between 2.5 and 25 μm; and (3) far infrared (FIR) is defined as wavelengths between 25 and 1000 μm. When a substance is exposed to NIR light from a light source, the infrared-active molecular bonds interact with the light to produce NIR spectrum absorption. The absorption of molecules in the infrared spectral region results from changes in the vibrational or rotational state or transitions between energy levels. Energy transitions include fundamental frequency transitions (corresponding to molecular vibrational state transitions between adjacent energy levels), double frequency transitions (corresponding to molecular vibrational state transitions that are separated by one or more energy levels), and combined frequency transitions (corresponding to the simultaneous transition of the energy levels of the two vibrational states of the molecule). All near-infrared absorption bands are multiplied and combined with the frequency of the fundamental mid-infrared absorption band (2000∼$4000\;{\mathrm{cm}}^{-1}$). Among them, the hydrogen-containing group X-H (such as C-H, O-H, N-H, etc.) is the dominant group. There is also information regarding other groups (such as C=C, C=O, etc.) but their intensity is weak. These groups are important organic matter constituents, and the NIR absorption wavelengths and intensities of the different groups or the same group in different chemical environments are significantly distinct. NIR spectroscopy contains a wealth of structure and composition information, which is excellent for evaluating the chemical and physical characteristics of substances.

An IR spectrum is a two-dimensional plot of the IR absorption values and the corresponding IR wavelengths. IR spectra contain three distinct types of peaks: (1) fundamental; (2) overtone; and (3) combination [1]. The fact that NIR spectra contain all three peak types but MIR spectra only contain the fundamental peak is the primary distinction between NIR spectra and MIR spectra. The molecular covalent bonds can be identified using the absorbed IR wavelengths because MIR spectra are straightforward and each molecular covalent bond is represented by a distinct group of IR wavelengths. In contrast, it is difficult to directly identify the molecular covalent bond in NIR spectra because each type of molecular covalent bond can be represented by a combination of the three peak types. Since the FIR electromagnetic signal has low energy, designing light sources and detectors can be challenging. Typically, increasing the energy of the FIR light source would result in an increase in temperature and require special materials to handle the temperatures [2]. The majority of this work was concentrated on MIR due to the challenges of FIR and NIR. NIR is used with data analysis algorithms that learn the relationship between the sample composition and NIR spectra, as opposed to MIR, where the sample composition can be determined by visually inspecting the peaks in the MIR spectra [3,4].

The composition of a sample, such as its protein, fat, vitamin, and fiber content, can be used to determine its quality. The food industry can use this data to identify premium food items. Similarly, the health sector can use the composition of a sample to determine the malignancy of a tumor or the agriculture sector can use it to assess the quality of manure.

To the best of our knowledge, this is the first review paper that focuses on the topic of machine learning for infrared spectroscopy. Although the majority of review articles investigate the broader topic of artificial intelligence (AI) for photonics, the coverage of machine learning algorithms used in infrared spectroscopy is relatively limited.

## 2. Machine Learning-Based NIR Spectroscopy Analysis System

**Figure 1:**
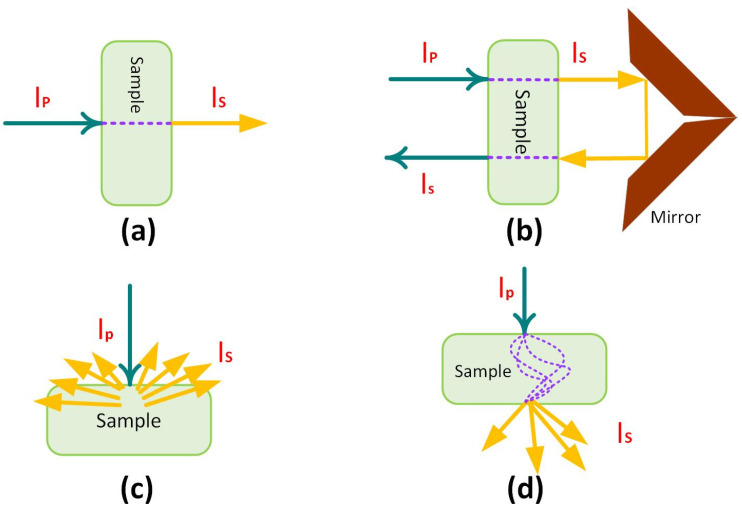
(**a**) Transmittance measurement mode, which is used with gases and semi-solids placed in a cuvette; (**b**) transflectance measurement mode, which is used with semi-solids without a cuvette; (**c**) diffuse reflectance measurement mode, which is used with solids where the measurement is taken from the NIR incidence; (**d**) transmittance through a scattering medium.

NIR spectroscopy is underpinned by three pillars: (1) fundamentals; (2) instruments; and (3) data analysis. As illustrated in Figure 1a–d, the fundamentals are the different measurement modes available in NIR: (a) transmittance; (b) transflectance; (c) diffuse reflectance; and (d) transmittance through a scattering medium [5].

The sample material determines the measurement mode used to produce the spectra. Transmittance mode is used for gases, liquids, or semi-solid samples, where the samples are placed in cuvettes and NIR is applied on one side and NIR transmittance is measured on the other. Without placing the sample in a cuvette, transflectance mode is used for semi-solid samples. In this mode, the sample is treated with NIR on one side, which penetrates the sample and is reflected through the sample using a stainless steel or gold reflector to measure the NIR transmittance. Hence, in transflectance mode, the light path is twice as long as that in transmittance mode. Diffuse reflectance mode is used for solid samples where NIR is applied on one side of the sample and the NIR scattering and absorption are measured. Interactance mode is applied to solid samples, where the absorption measurement is performed at a greater distance from the NIR incidence. Therefore, these absorption measurements are not affected by the NIR incidence signal but they may be affected by ambient NIR signals. In contrast, transflectance and diffuse reflectance measurement modes find the composition in the surface of the sample.

As there are different types of NIR instruments, the choice of which one to use depends on the application requirements, cost, signal-to-noise ratio, and measurement speed. NIR instruments can be classified as follows: (1) light-emitting diode (LED) [6]; (2) acousto-optic tunable filters (AOTF) [7]; (3) dispersive optics [8]; and (4) Fourier transform [9]. The least expensive instruments are those that use LEDs, and each LED produces a distinct NIR wavelength. AOTF-based instruments are fast as they do not contain any moving parts. AOTF-based instruments generate NIR of different wavelengths with a crystal made of TeO_2_, radio frequencies (RFs), and polychromatic light. The crystal adjusts the refractive index of the crystal using the RF signal to change the wavelength of the polychromatic light to the desired value. A reflective concave grating, which is used in dispersive optics-based instruments, shifts the wavelength of the polychromatic light. The first generation of NIR spectrometers employed dispersive optics, which are incapable of accurately producing NIR wavelengths. An interferometer and the Fourier transform are used in Fourier transform-based instruments to split the polychromatic light into NIR waves of various wavelengths. The main advantage of Fourier transform-based instruments is that they have a low signal-to-noise ratio. The advantages and disadvantages of the various NIR instrument types are compiled in Table 1.

Data analysis is the pillar that maps the NIR absorption or transmittance values to the desired sample properties. In this review article, we primarily highlight research on data analysis using machine learning.

**Figure 2:**
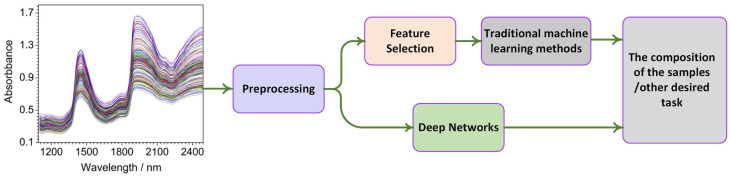
Architecture of machine learning for NIR spectroscopy.

As illustrated in Figure 2, ML algorithms map the NIR absorption values to the desired output. ML algorithms include training and testing phases. ML algorithms learn model parameters during the training stage using the light absorption values as inputs and the desired outcome as outputs. Based on the provided light absorption values, ML algorithms predict the desired outcome during the testing phase.

ML algorithms can be categorized as traditional machine learning methods and deep network architectures. Traditional machine learning methods have few or no hidden layers, such as partial least squares (PLS), K-nearest neighbor (KNN), principal component analysis (PCA), etc., whereas deep network architectures have multiple hidden layers such as AlexNet, GoogLeNet, etc. Traditional machine learning methods require an expert to engineer suitable features, whereas deep network architectures use raw features. In contrast to deep network architectures, the performance of traditional machine learning methods depends on the engineered features. As a result, deep network architectures are becoming more prevalent, as an expert is not required to engineer features.

Traditional machine learning methods employ feature selection algorithms to find interesting features. As a result, traditional machine learning methods usually take the form of a pipeline architecture, where feature learning is used to select interesting features followed by regressors or classifiers. In contrast, deep architectures have many hidden layers that are trained end to end including specialized layers such as convolution layers to learn local feature patterns and recurrent layers to learn the temporal information of the input data. Therefore, deep learning architectures generally outperform traditional machine learning methods when there are large numbers of training samples. Deep network architectures, however, frequently encounter overfitting issues and have high computational costs during the training phase when there are few training samples. Traditional machine learning methods can overcome the shortcomings of deep network architectures with insufficient data. In this scenario with limited data, deep network architectures augmented with regularization and dropout techniques are preferable to traditional machine learning methods.

## 3. Public Datasets

The methods used to collect the data and number of samples from freely accessible datasets are both described in this section. Publicly accessible datasets are available for a variety of uses including identifying tumors and analyzing soil, food, pharmaceuticals, and wood.

The Swedish soil dataset [10] provides data on the infrared spectroscopy absorption values of soil organic matter. The data were collected from soil plots located in Sweden, where each plot had a size of 120 × 120 cm. To produce soil organic matter, six different methods were used on six plots for a total of 36 plots. A total of 108 samples were collected, with one sample of organic matter taken from a depth of 0∼5 cm and two samples from a depth of 5∼10 cm. Roots and rocks were removed from the samples that were then manually homogenized for 15 min before being dried in an oven at 70 °C. Between the wavelengths of 400 and 2500 nm at 2 nm intervals, the infrared spectroscopy absorption values were recorded. The dataset is available at http://www.models.life.ku.dk/NIRsoil (accessed on 18 November 2022).

The corn dataset contains recordings of the infrared spectroscopy absorption values of corn. The absorption values were taken from a range of 1100∼2498 nm at intervals of 2 nm and their corresponding moisture, oil, protein, and starch values were recorded. There are 80 samples and 700 features in total in the dataset and it is available at http://www.eigenvector.com/data/Corn/index.html (accessed on 18 November 2022).

The tablet dataset contains recordings of the infrared spectroscopy absorption values of 654 tablets. The absorption values were taken from a range of 600∼1898 nm at intervals of 2 nm and their corresponding tablet ingredients API tramadol, plus talc, ethyl cellulose, and stearyl alcohol were recorded. The dataset is available at https://eigenvector.com/wp-content/uploads/2019/06/nir_shootout_2002.mat_.zip (accessed on 18 November 2022).

The melamine–formaldehyde (MF) dataset [11] contains recordings of the infrared spectroscopy absorption values of different chemical mixtures used in the polymerization process. The polymerization process generates different types of plastics. The absorption values were taken from a range of 3900∼11,000 cm at intervals of 1 cm from four chemical mixtures. Multiple readings were taken from each mixture, which yielded 1413 samples in total. The dataset is available by request to the authors of [11].

The soil dataset contains recordings of the infrared spectroscopy absorption values of different soil samples to identify fertility. The absorption values were taken from a range of 1000∼2500 nm from 40 soil samples and the fertility was measured by nitrogen (N), phosphorus (P), potassium (K), soil pH, magnesium (Mg), and calcium (Ca). The dataset is available at https://data.mendeley.com/datasets/h8mht3jsbz/1 (accessed on 18 November 2022).

The pleural effusion dataset contains the infrared spectroscopy absorption values of benign and malignant lung tissue samples. The absorption values were taken from a range of 4000∼10,000 ${\mathrm{cm}}^{-1}$ at intervals of 4 ${\mathrm{cm}}^{-1}$ from 82 tissue samples. There are 47 malignant tissues and 35 benign tissues. The data were divided into 62 and 20 samples for training and testing, respectively. The tissues were spun at 1600 g for 10 min at 4 °C and then stored at −80 °C before the absorption values were recorded. The dataset is available at https://www.ncbi.nlm.nih.gov/pmc/articles/PMC8093263/ (accessed on 18 November 2022).

The wood quality dataset [12] contains the infrared spectroscopy absorption values of wood with different defects: (1) knot; (2) decay; (3) bark (4) normal; (5) resin; (6) reaction; and (7) unsigned. The absorption values were taken from a range of 340∼2500 nm with four different light-detecting sensors. A total of 25 wood samples with diameters from 100 mm to 400 mm were collected from the wood cutting area and wrapped in aluminum before storing at −21 °C. These stored samples were transported to a lab where they were thawed to 15 °C before taking measurements. The measurements were taken at 36 locations, which resulted in a total of 1800 readings; 66% were used as training and the rest as testing. The dataset is available by request to the authors of [12].

The land use/cover area frame statistical survey (LUCAS) soil dataset [13] contains the infrared spectroscopy absorption values of soil samples and their respective soil properties: (1) clay; (2) silt and sand content; (3) coarse fragments; (4) pH; (5) organic carbon content; and (6) nitrogen. The absorption values were taken from a range of 400∼2500 nm at intervals of 0.5 nm. There are in total 19,019 samples, where 75% were used for training and 25% were used for testing. The dataset is available at https://esdac.jrc.ec.europa.eu/content/lucas-2009-topsoil-data#tabs-0-description=0 (accessed on 18 November 2022).

The chicken meat dataset [14,15] contains the infrared spectroscopy absorption values of chicken samples and their corresponding quality labels. Slaughtered chicken breast fillets were selected by an experienced analyst and there was a large variation in quality. A total of 158 samples within 5 h of slaughtering were transported under refrigerated conditions to the lab, where the central part of each sample was carefully trimmed with a surgical scalpel to fit into a sample cell. Subsequently, the samples were minced using a kitchen chopper for 10 s and the infrared spectroscopy absorption values from the wavelength range of 400∼2500 nm were collected. The quality labels were determined by the pH values and the colors, which were (1) pale; (2) pale, soft, and exudative; (3) dark, firm, and dry; and (4) normal. The dataset is available by request to the authors of [14,15].

The manure dataset [16] contains the infrared spectroscopy absorption values of manure samples and their corresponding properties: (1) dry matter; (2) ammonium nitrogen; (3) nitrogen; (4) calcium oxide; (5) potassium oxide; (6) magnesium oxide; (7) phosphorus pentoxide; and (8) the type of manure. A total of 332 manure samples were collected from France and Reunion island, where 196 were cattle samples and 136 were poultry samples. The samples were frozen after collection and homogenized by crushing them in a blender cutter. These samples were then dried for 4 days at 40 °C in a convection oven. The infrared spectroscopy absorption values were taken from three spectrometers, two of which had detection wavelength of 400∼2500 nm, whereas the third spectrometer had a detection wavelength of 4000∼12,500 cm. The dataset is available at https://doi.org.remotexs.ntu.edu.sg/10.15454/JIGO8R (accessed on 18 November 2022).

## 4. Data Preprocessing

Data preprocessing methods, whose aims are to separate the signal from the noise and reduce the signal-to-noise ratio, play a crucial role in the success of NIRS-based target sample composition estimation. In this section, we provide an overview of the most well-liked signal preprocessing algorithms that have been applied to NIR absorption spectroscopy signals in the past.

The Beer–Lambert law states that the absorption of NIR depends on the NIR path length, molecular absorptivity, and concentration of the sample. The molecular absorptivity and NIR path length are typically constant, and the NIR absorption is proportional to the sample concentration. However, the NIR scattering caused by sample particle distribution alters particle size, sample density, sample shape, path length, and molecular absorptivity. As a result, samples with the same concentrations would have different NIR spectra. These differences in NIR spectra are reflected in the additive bias noise for all NIR spectra, multiplicative bias noise for all NIR spectra, and additive and multiplicative bias noise in a specific wavelength in the NIR spectra.

Due to the fact that some machine learning algorithms perform poorly with noisy data, preprocessing aims to eliminate additive and multiplicative bias noise. The prevalent data preprocessing-related functions in NIR are (1) mean centering [17]; (2) standard normal variate (SNV) [18]; (3) multiplicative scatter correction (MSC) [18,19]; (4) extended multiplicative scatter correction (EMSC) [20]; (5) inverse scatter correction (ISC) [21]; (6) and Savitzky–Golay smoothing [22].

Detailed explanations of the preprocessing functions are available in [23] and the following subsections summarize the preprocessing functions used for NIR data.

### 4.1. Mean Centering and Standard Normal Variate (SNV)

Mean centering is the simplest approach to removing additive bias noise from all NIR spectra by calculating the mean and subtracting it. The SNV takes the mean centering further by removing both the additive and multiplicative bias noise from all NIR spectra by calculating the mean and variance, followed by subtracting the mean and dividing by the variance; both the mean centering and SNV assume that the additive bias noise can be approximated by the mean of the data and the multiplicative bias noise can be approximated by the variance of the data. Mean centering has been investigated with other preprocessing functions on six NIR datasets and found to be suitable if there are few training samples [17]. Sesame seed protein content was investigated using the SNV and it was found to be useful [18].

### 4.2. Multiplicative Scatter Correction (MSC)

MSC assumes that the noise can be described by a multiplicative and additive bias. This bias can be determined by comparing a noise-free reference NIR with each NIR data sample. MSC is applied by iteratively fitting the reference NIR sample with linear regression to each NIR data sample, followed by subtracting linear regression intercept and dividing the linear regression slope from the NIR data sample; the mean or median of all the data samples in the NIR data serves as an approximation for the reference NIR. MSC has been demonstrated to be helpful in determining the amount of protein in sesame seeds [18].

### 4.3. Extended Multiplicative Scatter Correction (EMSC)

EMSC extends MSC by removing nonlinear noise and the nonlinear noise sources are defined by an expert such as the path length correction terms and chemical constituents of the sample. EMSC iteratively calculates the linear and nonlinear multiplicative and additive noise for each NIR data sample based on a reference signal. The reference NIR is approximated by the mean or median of all the data samples in the NIR data. The effect of the path length in gasses can be effectively eliminated by EMSC [20].

### 4.4. Inverse Scatter Correction (ISC)

In contrast to MSC, ISC inverts the regressors for the reference NIR sample and dependent variables NIR data sample of MSC when applying linear regression to determine the multiplicative and additive bias noise. Furthermore, the reference signal of ISC is a known noise-free NIR data sample. ISC was shown to be an effective preprocessing function in eight food NIR datasets [21].

#### Savitzky–Golay

Savitzky–Golay is a filter that smooths the NIR signal by removing high-frequency noise. Savitzky–Golay performs polynomial regression on windowed NIR data samples. The amount of smoothing is thus determined by the window size and number of polynomials. This process is formulated as a convolutional operation, where the kernel represents the polynomial regression weights. Savitzky–Golay is typically used for NIR data that show a significant effect on the path length due to different particle sizes such as in soils [24].

### 4.5. Discussion

The NIR data are shaped during preprocessing to eliminate path-length differences and molecular absorptivity-related variability. The preprocessing functions shape NIR data differently, as they determine additive and multiplicative bias noise differently. Hence, the preprocessing functions should be carefully selected based on the application. For example, solid samples tend to have different path lengths than gas samples due to scattering caused by different particle sizes. Since gas samples have less variability than solid samples, a simple preprocessing step would suffice.

To find the best preprocessing function, experiments were conducted on 13 datasets of foods, liquids, and plants [25,26]. The results revealed that Savitzky–Golay smoothing with SNV preprocessing performed better than other preprocessing functions [25]. Savitzky–Golay smoothing outperformed other preprocessing functions according to the experiments on the soil spectra data that were conducted to determine the best preprocessing function [26]. However, it should be noted that Savitzky–Golay smoothing is computationally expensive.

## 5. Feature Selection

Feature selection has been shown to be an effective and efficient data preprocessing technique for preparing data (especially high-dimensional data) for a variety of data mining and machine learning problems. Finding the most consistent, pertinent, and non-redundant subset of features from the feature vector is the aim of feature selection. It lessens not only model complexity and training time but also the risk of overfitting. Feature selection algorithms can efficiently reduce the dimension of spectral data and remove redundant information from the spectrum. Here, we compile a brief overview of the most popular feature selection methods for NIR absorption spectroscopy as reported in the literature.

With regard to absorption values at particular wavelengths, NIR spectra data describe the composition of a sample. Contrary to expectation, noise causes the NIR spectra data to contain more peaks than expected, each of which corresponds to a different wavelength. As noise impairs the performance of machine learning algorithms, feature selection aims to identify the crucial features that describe the composition of a sample.

**Figure 3:**
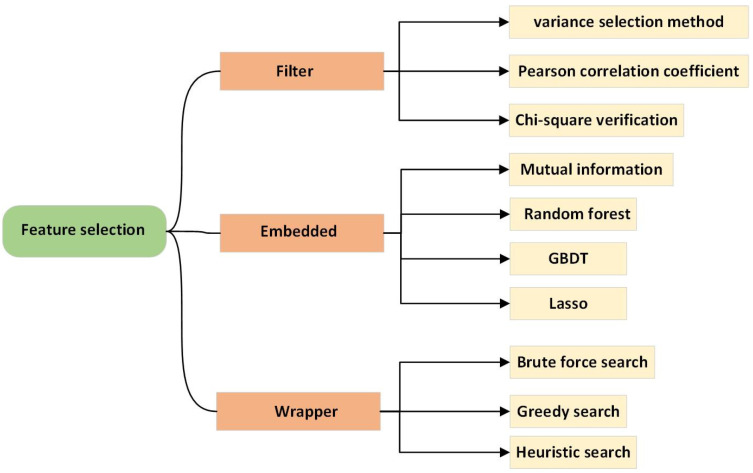
Classification of feature selection methods.

Feature selection algorithms are categorized as filter, wrapper, and embedded approaches. The classification diagram is depicted in Figure 3. The primary distinction between the three types of algorithms is how the learning algorithm is used to analyze and choose features.

The significance of the features is determined by filter-based approaches using a function such as statistics, distance, or similarity. As a result, features selected using filter-based approaches do not overfit and are ranked according to importance. Filter-based algorithms include covariance selection, minimal redundancy maximal relevance (mRMR) [27], and correlation-based feature selection (CFS) [14]. However, the filter-based approaches do not account for the generalizability of selected characteristics.

Wrapper-based approaches select features based on their generalization capability. Hence, wrapper-based features tend to overfit and most of the feature selection approaches in NIR use this approach. The exhaustive search required by wrapper-based methods to prevent overfitting also makes them computationally expensive. Wrapper-based algorithms used in NIR include particle swarm optimization (PSO) and binary particle swarm optimization (BPSO) [28], genetic algorithms (GA) [29,30,31,32,33], variable combination population analysis (VCPA) [32,34], the variable iterative space shrinkage approach (VISSA) [35], bootstrapping soft shrinkage (BOSS) [36], iteratively retaining informative variables (IRIV) [32], competitive adaptive reweighted sampling (CARS) [37,38,39], the successive projection algorithm (SPA) [40], uninformative variable elimination (UVE) [41], Monte Carlo uninformative variable elimination (MCUVE) [35], partial least squares feature selection approaches [42], the randomization test (RT) [43], variable importance in the projection (VIP) [44], and the jackknife procedure.

Embedded approaches consist of a model learning term that evaluates the generalization capability of selected features and a feature selection term to select features. Embedded approaches jointly optimize both terms and require fewer computational resources than wrapper-based approaches because they do not require the learning of multiple models. The least absolute shrinkage and selection operator (LASSO) [45,46] and elastic-net [47] algorithms are both examples of embedded approaches.

### 5.1. Particle Swarm Optimization (PSO) and Binary Particle Swarm Optimization (BPSO)

PSO is an evolutionary computational technology that evolved from research into bird predation behavior. The basic idea behind the particle swarm optimization algorithm is to find the best solution through group cooperation and information sharing. Birds are abstracted as particles (points) in an *N*-dimensional space without mass or volume. The position of a particle in the *N*-dimensional space is expressed as a vector $X_{i}=\left(x_{1},x_{2},\ldots ,x_{N}\right)$, and the flight speed is expressed as a vector $V_{i}=\left(v_{1},v_{2},\ldots ,v_{N}\right)$. Each particle has a fitness value that is determined by the objective function. Additionally, each particle is aware of the best position ($p_{best}$) discovered thus far and the current position ($X_{i}$). This could be considered the particle’s personal flight experience. In addition, each particle is aware of the best position ($g_{b}est$) discovered to date by each particle in the whole group ($g_{best}$ is the best value in $p_{best}$), which can be regarded as the experience of the particle companion. Particles determine their next movement based on their and their peers’ best experiences.

The following are the update rules:

(1) $$v_{i}=v_{i}+c_{1}*rand\left(\right)*\left(P_{best_{i}}-x_{i}\right)+c_{2}*rand\left(\right)*\left(g_{best_{i}}-x_{i}\right)$$

(2) $$x_{id}^{k}=x_{id}^{k-1}+v_{id}^{k-1}$$

where $v_{id}^{k-1}$ denotes the *d*-th dimension vector of the (k−1)-th iteration’s flight velocity vector for particle i; $x_{id}^{k}$ the *d*-th dimension vector of the position vector of particle *i* in the *k*-th iteration; $c_{1}$ and $c_{2}$ the learning factors that are usually set to 2; rand() a random number between 0 and 1; $x_{i}$ the current location of the particle; and $v_{i}$ the particle’s speed.

In contrast to PSO, which focuses on continuous real-value problems, BPSO prioritizes discrete-space constraint problems [28]. The BPSO algorithm is based on the discrete particle swarm algorithm, and it is agreed that the position vector and velocity vector are composed primarily of 0 and 1 values, respectively. Although BPSO has good global search capabilities, it cannot converge to the global optimal value. Moreover, as the algorithm searches iteratively, the randomness grows stronger and stronger and it lacks local search capabilities in the later stages. In the study in [48], four soy sauce quality parameters were determined using Vis-NIR techniques in conjunction with variable selection using a simple modified particle swarm optimization (PSO) algorithm. The findings demonstrated that the application of variable selection based on a modified PSO optimization algorithm not only simplified the models but also significantly improved the quality of the models in terms of accuracy and reliability.

### 5.2. Genetic Algorithms (GAs)

A GA is stochastic and based on biological evolution and genetics. A GA consists of five steps: (1) initialization; (2) fitness; (3) selection; (4) crossover; and (5) mutation. Different sets of features are randomly chosen during the initialization step. The fitness step evaluates each set of features through cross-validation and ranks them based on cross-validation accuracy. Two sets of features are chosen in the selection step based on rank and non-uniform random selection probability (NRSP). NRSP has a high probability value for a set of features with a higher rank and a low probability value for a set of features with a low rank. A crossover step creates a new set of features by randomly selecting features in a non-overlapping manner from the two selected sets of features in the selection step. A feature from the freshly produced set of features is randomly selected or deselected during the mutation process. Steps 2 through 5 are repeated until a stopping criterion is met such as the number of iterations or the desired accuracy of cross-validation. GAs have been used to determine food quality [32,33], the quality of soil [29], document dating [30], and ailments using blood plasma [31]. The study in [49] proposed a nondestructive method for determining the internal quality of apples using a contactless NIR spectrometer and genetic algorithm for model optimization. The performance of the contactless system was enhanced by 30% as a result of model optimization using genetic algorithms, bringing it closer to the performance of the models from the multipurpose analyzer.

### 5.3. Covariance Selection

The covariance selection method chooses features by calculating the correlation matrix between the input and output data, then choosing input data features that have a strong correlation with output data features. To address the fact that visible and near-infrared (Vis-NIR) spectra are produced by the combination of many low-resolution features, the spectral variables are highly correlated, which causes difficulties in the selection of the most appropriate variable for a given application. This study proposes the application-dedicated selection of filters (ADSF), which can choose the most relevant subset of filters from any predefined shapes by maximizing covariance and using orthogonal projection. The ADSF acts as a regularization process, resulting in a selection that should be resistant to overfitting even in the context of a small sample size [50].

### 5.4. Variable Combination Population Analysis (VCPA)

VCPA consists of three steps: (1) binary matrix sampling (BMS); (2) model population analysis (MPA); and (3) feature selection. BMS is a random sampling matrix that includes one and zero values to uniformly and equally likely select and deselect features, respectively. The MPA step calculates the cross-validation accuracy of the sampled features using BMS. The frequency of each feature being selected from the top 10% of the cross-validation accuracy is then calculated. Finally, an exponential function is used in the feature selection step to determine the number of features to retain based on the frequency with which each feature is selected. These steps are repeated until the stopping criteria, such as the number of maximum iterations or the desired cross-validation accuracy, are met. To achieve the rapid detection of the bacteria food-borne pathogen (Escherichia coli O157 and Staphylococcus aureus) contamination of fresh longissimus pork muscles, in the study in [32], visible near-infrared (V-NIR) hyperspectral imaging along with PLSR and VCPA algorithms were proposed for the prediction and quantification of Escherichia coli O157: H7 and Staphylococcus aureus. The results demonstrate that the updated VCPA step is a very effective way to eliminate irrelevant variables.

### 5.5. Variable Iterative Space Shrinkage Approach (VISSA)

VISSA randomly samples features using BMS sampling and then uses PLS models to calculate the cross-validation accuracy for each randomly sampled feature. The top 5% of PLS models are then chosen, and features are chosen using the high-valued model coefficients. To determine whether the chosen features are the best, a PLS model is then created with the chosen features, and its cross-validation accuracy is compared to the cross-validation accuracy of the prior model. If the PLS model’s cross-validation accuracy with the selected features is less accurate than without feature selection, BMS is applied once more to choose features. VISSA repeats the aforementioned steps until the stopping criteria are met in order to further optimize the optimal features that have been chosen. The origin of apples was identified using a method combining a variable iterative space shrinkage approach with stepwise regression (VISSA-SR), which obtained the characteristic wavelength effectively and reduced the modeling process’s operating time [35].

### 5.6. Bootstrapping Soft Shrinkage (BOSS)

The correlation between the input and output data is used by BOSS to choose features. In contrast to covariance selection, which only considers global feature importance, BOSS determines the optimal features by considering both local and global feature importance. BOSS consists of three steps: (1) sampling; (2) MPA; and (3) feature selection. First, BOSS randomly samples the features of the input data, selecting only 63.2% of the features without repetition. The cross-validation accuracy of each feature set is determined in the MPA step using PLS. The absolute weight vector of each PLS model is normalized during the feature selection step, and the normalized weight vectors of each model are then added to determine the likelihood of the feature being selected. If the added weight is high or low, the likelihood of selecting a feature will be high. BOSS therefore assumes that a high absolute weight value denotes an interesting feature. Finally, BOSS samples the features based on the probability of feature selection to choose 63.2% of the features using weighted bootstrap sampling (WBS). The process iterates until a stopping point is reached such as the maximum number of iterations, the minimum number of optimal features, or the predetermined cross-validation accuracy. The determination of the adulteration content in extra virgin olive oil using FT-NIR spectroscopy combined with the BOSS–PLS algorithm was proposed in [36]. The results obtained demonstrate the superiority of the BOSS algorithm in the selection of instructive wavenumbers.

### 5.7. Iteratively Retaining Informative Variables (IRIV)

IRIV is an exhaustive feature selection approach that uses the Mann–Whitney U test to determine the optimal features. IRIV first uses BMS to create a set of features and select a feature of interest in the feature set. The feature of interest is added to the feature set to calculate the mean cross-validation accuracy, and the feature is excluded from the feature set to calculate the other mean cross-validation accuracy. To determine whether the feature of interest is useful, the Mann–Whitney U test is finally run on the two mean cross-validation values. Iteratively selecting the feature of interest from the first to last features is computationally expensive. Therefore, IRIV is frequently combined with other feature selection algorithms. A feature selection algorithm such as VCPA is used to pick out a select few features from the input data and the selected features are then subjected to IRIV to further reduce the number of features. Pork quality was assessed using IRIV in [32] and the purpose of the study was to assess whether it is feasible to create an enhanced and effective reduced spectrum model for quantitatively tracking food-borne pathogens. The results of the experiment demonstrate that, in comparison to other methods, variable combination population analysis combined with a genetic algorithm (VCPA-GA) and variable combination population analysis combined with iteratively retaining informative variables (VCPA-IRIV) can significantly increase the model’s predictive effectiveness.

### 5.8. Competitive Adaptive Reweighted Sampling (CARS)

The CARS algorithm determines the optimal features by assuming that the optimal features are represented by large absolute PLS regression weights. CARS first calculates the regression weights using PLS and then normalizes the regression weights. The exponential decreasing function (EDF) is used to determine the number of features to be retained based on the values of the normalized regression weights. To further choose features based on the normalized regression weights, adaptive reweighted sampling is then used. A high regression weight value denotes a high likelihood that the feature will be chosen, whereas a low regression weight value denotes a low likelihood that the feature will be chosen. These steps are iteratively repeated until the stopping criteria, such as the number of maximum iterations or the desired cross-validation accuracy, are reached. CARS was used to determine the quality of oilseed [37], rice [38], and seeds [39]. To find the rice-grain moisture NIR spectroscopy [38], the partial least squares (PLS) and competitive adaptive reweighted squares (CARS) models were used to model and analyze the spectral data. The findings demonstrate the effectiveness of the CARS feature selection algorithm.

### 5.9. Successive Projection Algorithm (SPA)

The SPA is a forward feature selection method that builds on one feature at a time until the desired cross-validation accuracy is achieved. PLS is used iteratively by the SPA to calculate the cross-validation accuracy from the first feature to the feature where it stops increasing. As a result, the SPA combines PLS and forward feature selection into a single algorithm. The SPA was used to determine the quality of grape seed oil [40]. The study in [51] proposed an alternative analytical technique for determining the fat content of commercial chicken hamburgers based on near-infrared (NIR) spectroscopy and the SPA for interval selection in partial least squares regression (iSPA-PLS), which outperformed full-spectrum PLS and iPLS in terms of predictive performance.

### 5.10. Uninformative Variable Elimination (UVE)

The UVE feature selection algorithm removes noisy features. PLS is initially used to determine the leave-one-out cross-validation accuracy and model coefficient stability values by dividing the mean of each coefficient by the standard deviation of the coefficient. The accuracy of the leave-one-out cross-validation is then calculated by building a new model on the noisy data after adding uniform random noise with a noise level of $10^{-10}$. The coefficient stability values for the model made with noisy data were then calculated and features larger than the coefficient stability values for the model made with noise-free data were eliminated. It is possible to combine UVE and the SPA to remove noisy and uncorrelated features. UVE and MCUVE were used to determine the adulteration of virgin olive oil [41]. The study in [52] proposed the UVE-SPA method, a successive projection algorithm (SPA) combined with uninformative variable elimination (UVE), which was effectively used for variable selection in the NIR spectroscopic analysis of nicotine in tobacco lamina and active pharmaceutical ingredients in intact tablets.

### 5.11. Monte Carlo Uninformative Variable Elimination (MCUVE)

MCUVE is an extension of UVE that lowers computational complexity by replacing leave-one-out cross-validation with random leave-one-out cross-validation. The process of calculating the coefficient stability values of noisy data is also skipped by MCUVE by eliminating features with low coefficient stability values. To predict pH in lime concretion black soil, the study in [53] used CWT to preprocess the soil spectra, followed by ELM combined with four spectral variable selection methods, GA, SPA, MCUVE, and CARS, and the full spectrum. According to the results of the experiment, the MCUVE feature selection algorithm had the lowest residual prediction deviation.

### 5.12. Randomization Test (RT)

The RT is a two-step process for feature selection. The input data are used to learn a model and the output data are randomly permuted in the first step. This process is repeated numerous times to learn various random models. With the input and output data, the model is learned in the second step. Then, features that had smaller normal model coefficient values than random model coefficient values were removed. Compared to CARS and UVE, the RT is a more efficient method for feature selection in NIR datasets [54] despite being computationally intensive due to its random permutation. In the study in [55], a novel method known as RT-PLS for wavelength selection in NIR spectral analysis was proposed based on the randomization test. In the suggested approach, a statistic can assess the significance of the variables in a spectrum.

### 5.13. Variable Importance in the Projection (VIP)

VIP uses PLS coefficients to select features. To achieve this, VIP first learns the model of the input and output data before determining the importance of the features based on the coefficients weighted by the output data. Weighted coefficients with values higher than 1 are selected. VIP was used to determine the quality of nursery plants. Three scientifically recognized indicators—the vector of the regression coefficients, the selectivity ratio, and VIP—were used to analyze the most crucial variables to distinguish between these varieties [44]. The experimental results show that VIP can select the key variables to distinguish between these varieties.

### 5.14. Jackknife Procedure

The jackknife feature selection procedure generates PLS models equal in number to the number of training samples by removing one sample at a time. The ratio between the model coefficients and the standard deviation of the model coefficients is then calculated for each feature. A high ratio value denotes the usefulness of a particular feature. The study in [56] proposed analyzing NIR spectroscopy data using a functional approach. They used an approach based on the leave-one-out jackknife procedure technique to assess the variance in such estimates.

### 5.15. Minimal Redundancy Maximal Relevance (mRMR)

The mRMR algorithm minimizes redundancy and maximizes the relevance between features. Relevance describes the highest correlation between the selected features in the input data and output data and redundancy describes the smallest correlation between those features in the input data. Redundancy can be determined for discrete and continuous data using mutual information and Pearson’s coefficient, respectively. Similarly, mutual information and F statistics are used to determine the relevance of the discrete and continuous data, respectively. In order to select a feature with the least amount of redundancy and the greatest amount of relevance with respect to the selected features in the pool, mRMR iteratively selects the features from the input data that are not included in the pool. mRMR reiterates this procedure until the stopping criteria such as the maximum number of features are reached. The study in [57] investigated a novel feature selection technique, the mRMR, for choosing the best wavelengths from the visible to near-infrared spectra of the hyperspectral imaging data for classifying foreign matter in cotton. The experimental results show that the selected wavelengths are compatible with a variety of cotton foreign matter classifiers.

### 5.16. Correlation-Based Feature Selection (CFS)

The CFS method seeks to maximize relevancy between features while minimizing redundancy. In contrast to mRMR, the CFS stopping criteria are based on the shift in the relevance-to-redundancy ratio that occurs when a new feature is chosen. This means that CFS stops selecting features if the change in the ratio is marginally smaller than a predefined value. In the greedy feature selection method used by CFS, features are added to the pool if they produce the highest ratio of relevance to redundancy. Backtracking can be used to further improve the selection process after adding a feature to the pool by removing features one at a time and calculating the ratio between relevance and redundancy. Backtracking steps should be limited to lessen the computational complexity. CFS was used to determine the quality of chicken meat [14]. To achieve an efficient dimensionality reduction, correlation-based feature selection (CFS) was used to remove irrelevant and redundant information from the spectra.

### 5.17. LASSO and Elastic Net

LASSO consists of two terms, where one is the OLS term and the other is the $L_{1}$ weight penalization term. The OLS term evaluates the generalization capability of the selected features and the $L_{1}$ term selects the features by shrinking the weights representing the unimportant features to zero. LASSO was used to determine the quality of diesel in [45] and the goal of the study was to create a model for predicting diesel fuel parameters using data from a near-infrared spectroscopic analysis of the fuel. The results show that LASSO variable selection, followed by regression tree modeling, produced the best prediction accuracy.

Elastic net is LASSO with an extra $L_{2}$ weight penalization term. From a group of correlated features, LASSO will only select one feature. Elastic net resolves this shortcoming of LASSO with the aid of the extra $L_{2}$ penalization term. As a result, elastic net selects more features than LASSO and is better suited for multicollinear data such as NIR data [47].

### 5.18. Discussion

NIR spectra data contain a large number of features and low number of samples. Therefore, NIR spectra data are sparse because there are few data samples to cover this vast dimensional space, a problem known as the curse of dimensionality. The use of a features selection algorithm to reduce the number of features so that NIR spectra data are dense in low-dimensional spaces is a common approach to resolving this issue.

The composition of a sample is described by a set of particular wavelengths that are identified by feature selection. For instance, the amount of moisture in tea can be determined using wavelengths between 6694∼7293 cm and 7892∼8193 cm. These specific ranges were determined by applying a feature selection algorithm [28]. The fact that the first-order O-H stretching bond frequency doubles at 7143 cm [58] indicates that the feature selection algorithm has found potential ranges for the chosen amount of moisture. The number of features are decreased through feature selection, which also lowers the computational complexity of the machine learning models. Because of this, such machine learning models with low complexity can be used in portable NIR devices.

## 6. Traditional Machine Learning Methods for NIR

The machine learning architectures for NIR reported in the literature can be divided into two categories: traditional machine learning methods and deep network architectures. In a typical pipeline architecture based on traditional machine learning techniques, feature learning is used to select interesting features from the input data and then traditional machine learning techniques are used to model those features to produce the output data. Its limitation is the restricted generalization capability for complex classification problems due to the limited representation ability of complex functions in the case of limited samples and computing units. Applications of traditional machine learning methods to NIR spectroscopy that have been reported in the literature are compiled in this section. A summary of traditional machine learning methods in NIR is compiled in Table 2.

NIR data are affected by multicollinearity due to fundamental, overtone, and combination peaks. Multicollinearity is the redundant representation of the same component by multiple features such as the O-H bond in the sample. A sample should ideally have a single feature for each component. The ability of traditional machine learning algorithms to generalize is negatively impacted by the linear relationship between redundant features. By removing these redundant features, traditional machine learning methods for resolving multicollinearity in NIR can be used including (1) partial least squares (PLS) [59,60]; (2) extreme learning machines (ELM) [61,62,63]; (3) support vector regression (SVR) [64] and support vector machines (SVM) [65]; and (4) single-layer feed-forward neural networks (SLFN) [66].

### 6.1. Partial Least Square (PLS)

As a result of multicollinearity, NIR datasets lack full rank and cannot be modeled using ordinary least squares (OLS). PLS retains the input data features that have a strong correlation to the output data and learns a regression model between those features and the output data. In contrast to the random noise that PLS removes, extensions of PLS such as orthogonal partial least squares (OPLS) aim to remove the structured noise [67]. LASSO and PLS are combined for feature selection and NIR data analysis, respectively, in another variant known as sparse partial least squares (SPLS) [68]. A detailed explanation of PLS variants is available in [69]. The PLS method is one of the most frequently employed methods for analyzing NIR spectral data. For instance, partial least squares (PLS) regression was used in [70] to develop a calibration model for the prediction of the concentration of two antioxidants, Irganox 1010 and Irgafos 168, in high-density polyethylene. The findings show that PLS regression can be used to extract valuable chemical information from NIR spectral data.

### 6.2. Extreme Learning Machine (ELM)

The output of the ELM feature-embedding algorithm is a random sample of the features of the input data. In ELM, random sampling is accomplished by multiplying the input data with a random matrix that randomly scales and rotates the input data. If there are sufficient steps in random sampling, ELM feature embedding can learn the uncorrelated features. The number of hidden neurons in an ELM determines the number of random sampling steps. Therefore, more ELM hidden neurons are preferred. Finally, ELM develops a regression model between the output data and the ELM feature embedding. A detailed explanation of the variants of ELM can be found in [71]. The in [72] proposed a novel recognition method that employs a near-infrared (NIR) spectrometer and a multilayer–extreme learning machine (ML-ELM) algorithm to quickly and non-destructively identify the production regions of flue-cured tobacco leaves. The results show that different Yunnan tobacco-leaf-producing regions can be quickly, precisely, and non-destructively identified using a combination of NIR and ML-ELM.

**Table 2:** Summary of traditional machine learning methods used in NIR.

Ref. Publish Date	NIR Task	Models	Merits	Limitation
Apr. 2007, [58]	Wine	Principal component analysis (PCA)+partial least squares (PLS)	Can effectively calibrate.	The creation of NIR calibrations for wine compositional parameters was not the aim of this study.
May 2018, [73]	Olive oils	Partial least squares regression (PLSR)	The major and minor components of olive oils can be simply, quickly, and simultaneously quantified.	The performance of individual sterol form-prediction models was subpar.
Jun. 2017, [74]	α-tocopherol and total tocopherol contents	PLS and discriminant analysis (PLS-DA)	Quick and practical techniques used in the industry for sorting olive oils.	The number of samples were limited.
Mar. 2021, [75]	Moisture, protein, and fat in meat	Orthogonalization (SPORT)/orthogonalization (PORTO)+PLSR	Reduced the error and bias by up to 52% and 84%, respectively.	A combination of data from various scatter-correction techniques was required.
Apr. 2019, [39]	Rice-grain moisture	PLS+competitive adaptive reweighted squares (CARS)	Rapid determination of rice-grain moisture.	The results of stability and transitivity verification experiments for models were not provided.
Dec. 2019, [76]	Rice flour types	PLS-DA+support vector machines (SVM)	High level of accuracy.	The robustness of the model needs to be verified further.
May 2020, [77]	Multiple adulterations of flaxseed oil.	Orthogonal partial least squares–one-class partial least squares (OPLS-OCPLS)	Can effectively detect single, dual, or multiple adulterants with high accuracy; can rapidly detect multivariate adulteration of known targets.	The types of actual adulterated flaxseed oils were insufficient and the recognition accuracy of 95.8% still needs to be improved.
Aug. 2020, [78]	Milk powder	Multivariate curve resolution–alternating least squares (MCR-ALS)	Can correctly identify.	Inadequate milk samples were tainted with melamine and sucrose.
Dec. 2021, [79]	Hemoglobin concentration of blood	Monte Carlo+least absolute shrinkage and selection operator+extreme learning machine (MC-LASSO-ELM)	Better stability and the highest accuracy.	The model operation procedure was complicated and the MC results for a subset of samples had a direct impact on the results of the complete model.
Sep. 2017, [80]	Osteoarthritis	PCA+SVM+PLS	Demonstrated the capacity of NIR spectroscopy to monitor changes in the articular cartilage matrix.	The ability to assess the capacity of NIR spectroscopy to estimate collagen-related information was not provided.
Mar. 2020, [81]	Soil organic matter (SOM)	Savitzky–Golay (SG)+standard normal variate (SNV)+first derivative (FD)+PLSR	Rapid test; a simple and nondestructive analytical method.	The preprocessing procedure was complicated; preliminary experimental results.
Mar. 2017, [82]	Coffee	Genetic algorithm+SVM	A fast and effective method without the production of chemical wastes.	Sample selection was required.
Apr. 2022, [83]	Sulfur hexafluoride	GA-ELM	Higher prediction accuracy; operating efficiency; better stability; generalization performance.	It was challenging to effectively extract features using the GA algorithm.
Oct. 2015, [84]	Seed oil	MLR+SVR+ANN	Fast, simple, and lower prediction error.	Better wavelength selection methods for use as input signals should be addressed.
Jun. 2017, [33]	Acid value in peanut oil	GA-Si-PLS	Simultaneous and rapid measurement of acid value in peanut oil.	All of the algorithms compared were simple PLS-based algorithms.
Sep. 2017, [85]	The rancidity of perilla oil	ANN multivariate analysis methods	ANN models produced the best prediction results.	Only PCR and PLSR were used to compare the experimental results and the model’s parameters can be further optimized.
Sep. 2017, [86]	Oil, phenols, glucosinolates, and fatty acid content in the intact seeds of oilseed Brassica species	Modified partial least squares (MLPS)	Higher prediction accuracy.	The NIRS-based equation should be improved further by including samples from various environments with an even greater range of values.
Nov. 2017, [87]	Copaiba oils	PLSR	Fast; no sample preparation was required; reliability.	More algorithms must be compared to demonstrate the superiority of PLSR.
Jun. 2019, [36]	Olive Oil	BOSS-PLS	Rapid quantitative analysis.	The experimental samples were not diverse enough.
Nov. 2019, [6]	Sugar content estimation of citrus	Stepwise multiple linear regression (SMLR)	Higher detection efficiency.	Online citrus experiment detection was required.
Aug. 2018, [14]	Chicken meat	SVM; Decision trees	Avoided complex configurations or need for expertise in a particular technique.	Accuracy could be further improved.
Apr. 2016, [88]	Sesame seeds	Multi-elemental discriminant analysis	Classification accuracies of more than 90%.	Further seed sample analysis was necessary in order to increase the discrimination accuracy.

### 6.3. Support Vector Machine (SVM) and Support Vector Regression (SVR)

SVM is a classifier that learns a hyperplane that maximizes the difference between two classes. The hyperplane is computed by locating data points close to the class boundary; these data points are called support vectors (SV). A regressor called SVR learns a hyperplane based on two boundary planes that are equally spaced from it. Most data points to be learned are captured by the boundary planes. Multicollinearity is eliminated using a regularization term to remove the dependent data points in the SVM and SVR algorithms. The linear relationships between the input data and output data are modeled using SVM and SVR. SVM and SVR both model linear relationships between the input and output data. A kernel employs the inner product in a high-dimensional space to assess the similarity between data points. The hyperplane between the kernel and the output data is learned by the kernel SVM and SVR. A detailed explanation of SVM and SVR can be found in [89]. The work in [90] proposed using a combination of near-infrared (NIR) imaging spectroscopy and support vector machines (SVM) to detect meat and bone meal (MBM) in compound feeds. SVM performed significantly better than the other two stoichiometric PLS and ANN algorithms, with a significantly lower rate of false-positive detection.

### 6.4. Single-Layer Feed-Forward Network (SLFN)

SLFNs [66] are networks that have an output layer and one hidden layer. An SLFN’s hidden nodes can be neuron-like sigmoid hidden nodes or Fourier hidden nodes. An SLFN’s hidden- and output-layer weights are trained with back-propagation, which calculates the gradients of the network parameters at each layer, and use gradient descent to update the network parameters at each layer. The uncorrelated features of NIR datasets are learned using regularization. The study in [91] investigated the potential of combining an SLFN, which consisted of 451 neurons in the input layer, 1 to 30 neurons in the hidden layer, and a single neuron in the output layer, and near-infrared spectroscopy (NIRS) to predict sensory attributes. The results indicated that the optimized SLFN architecture applied to NIR spectra correctly classified all samples.

### 6.5. Decision Tree (DT) and Random Forest (RF)

A decision tree (DT) [92] is a non-parametric architecture that consists of nodes in a tree structure. The nodes consist of a decision rule based on input data that decides whether to transverse the right- or left-side nodes’ and the bottom nodes’ output data. A DT is used for both classification and regression tasks and the main advantage is that the algorithm is interpretable. Information gain is a popular method for building nodes in a tree that uses entropy to calculate the amount of information each feature in the input data retained before determining the output data. The study in [93] presented several examples of how DTs and their ensembles can be used in the analysis of NIR spectroscopic data both for regression and classification. The experimental finds demonstrate that the DT method is very efficient for the classification/discrimination of NIR data including multivariate datasets.

A random forest (RF) [94] is an ensemble of DTs, where each DT is created by a subset of the input data. As a result, each DT architecture is distinct from the others and decisions are made by a majority vote. However, for RF to function, the subsets of the input data used to create the individual trees should be uncorrelated. The same DT architecture could be produced by correlated subsets of the input data. The study in [95] proposed to determine the food dye indigotine in cream using near-infrared spectroscopy technology in conjunction with a random forest model. The experimental results presented in the study show for the first time that NIRS in conjunction with a random forest model is an effective tool for the quick and non-destructive prediction of indigotine pigment in cream.

### 6.6. Discussion

The investigation of different applications has been the primary research focus of NIR. NIR has been used extensively in applications where determining the quality of food is time consuming due to the use of intrusive approaches [14,33,38,73,74,75,76,96,97]. The adulteration of expensive food items has become widespread and NIR has been used to successfully determine adulteration levels [36,77,78,87,98,99,100]. As a low-cost quick method for identifying illnesses, NIR has also been used in health applications [79,80]. Due to the fact that the geographic location only provides a cursory indication of the quality of a food or mineral, NIR is able to determine the location of the production of these items [81,82,101]. In addition, NIR is also capable of determining wood quality [12], determining the age of polyvinyl chloride (PVC) [102], assessing water pollution [103], determining sulfur hexafloride concentrations [83], determining the adulteration of diesel blends [104], classifying types of fruits and vegetables [105], classifying types of seeds [106], and determining stem rot in oil palms [107].

Some research has investigated developing new algorithms for NIR and performing extensive hyperparameter selection to determine the efficacy of the proposed algorithms such as ensemble extreme learning machine (EELM) [108], boosting ELM [109]. Hyperparameter selection for SVM based on support vector (SV) and grid-based searches has been investigated for NIR so that the learned model is interpretable [110].

Data fusion has been used in NIR to combine information from different sensors that capture different information such as determining the adulteration level of honey using NIR and MIR [111], determining the contents of rice flour using NIR and MIR [112], determining rice storage life based on storage conditions using NIR and gas sensors [113], enhancing tea processing using NIR and computer vision [114], determining the quality of plant seeds using NIR and X-ray images [115], and determining the quality of fruits using two portable NIR instruments with different ranges [116].

## 7. Deep Architectures for NIR

Deep architectures are multilayered architectures that integrate classification or regression tasks end to end. They are capable of approximating complex functions by learning a deep nonlinear network structure, defining the distributed representation of the input data, and showcasing a potent capacity to learn the key properties of datasets from a limited number of test sets. Deep learning neural networks are able to automatically learn effective feature representations by applying nonlinear transformations to raw data features. The most common deep architectures and their applications in NIR spectroscopy are summarized in this section. A summary of deep architectures in NIR is compiled in Table 3.

The most common deep architectures include (1) stacked autoencoders (SAEs) [117]; (2) variational autoencoders (VAEs) [118]; (3) convolutional neural networks (CNNs) [119]; (4) CNNs with recurrent neural networks (RNNs) [120]; (5) multilayer–extreme learning machines (ML-ELMs) [121]; (6) local receptive field–extreme learning machines (LRF-ELMs) [122]; and (7) generative adversarial networks (GANs).

### 7.1. Stacked Autoencoder (SAE) and Variational Autoencoder (VAE)

An SAE consists of an encoder that learns a low-dimensional manifold of the input data and a decoder that reconstructs the input data. In reverse order, the decoder has the same number of layers and hidden neurons as the encoder. Being a multilayer network, an SAE converges to a local minimum. To improve convergence, a two-step learning approach is used. A single-layer autoencoder (AE) learns the encoder–decoder weights for each layer in the greedy layer-wise learning step before fine-tuning the SAE. As a result, the SAE divides the multilayer SAE learning into two phases: single-layer AE learning and multilayer AE fine-tuning. Wojciech et al. at the AGH University of Science and Technology employed three different models to evaluate soil and land: an SAE, a CNN, and the stack model, which consists of a collection of multilayer perceptron algorithms with two distinct methods for regression estimation that analyze the Vis-NIR spectral response signal [123]. Fu et al. proposed a stacked sparse autoencoder combined with a cuckoo search (CS)-optimized–support vector machine (SSAE-CS-SVM) for analyzing the near-infrared (NIR) hyperspectral data of maize seeds (871.61–1766.32 nm) to achieve maize seed variety identification [124].

**Table 3:** Summary of deep architectures used in NIR.

Ref. Publish Date	NIR Task	Models	Merits	Limitation
Dec. 2022, [125]	Bright-blue pigment in cream	Autoencoder–deep learning (AE-DN)	Lower calculation costs, high accuracy, and faster speed with samples undamaged.	Redundant signal preprocessing steps.
Sep. 2019, [126]	Aristolochic acids (AAS)	1D CNN	Without feature extraction, could effectively, nondestructively, and rapidly identify.	The experimental data sample was limited and no comparisons to other deep learning methods were made.
Jun. 2020, [127]	Drugs	CNN-based transfer learning	Higher classification accuracy with fewer training data.	Validation was performed with small experimental datasets; does not compare with state-of-the-art transfer learning models.
Aug. 2020, [128]	Salmon, tuna, and beef delicacies	CNN-based machine learning	With a shift-invariant feature, the variation caused by the use of multiple devices in a real-world setting can be minimized.	The types of freshness recognition must be expanded, real-world scene applicability must be improved, and recognition accuracy can still be improved.
Sep. 2021, [129]	Fresh fruit	Multi-output 1-dimensional convolutional neural network	Lower RMSE; easily adaptable to multi-response modeling by altering the output of the fully connected layers.	The use of transfer learning to process, update, and transfer a single model to integrate multiple responses was not discussed.
Jun. 2019, [130]	Soil	Convolutional neural network	Multitask learning ability; multidimensional input utilization; higher performance; interpretability of the important wavelength variables used to predict soil properties through sensitivity analysis.	Data hungry; many hyperparameters; requires more advanced computing hardware.
Nov. 2021, [131]	Tea	Standard normal variate (SNV)+TeaNet; SNV+TeaResNet; SNV+TeaMobilenet	A quick, non-intrusive, and environmentally friendly solution with 100% accuracy.	Various NIR data types necessitated the selection of the best data preprocessing method.
May 2021, [132]	Dried mango	Chemometric approaches + DL models	Improved the predictive performance of DL models; achieved the lowest RMSEP.	The use of large datasets is required.
Dec. 2020, [13]	Soil total nitrogen (STN) content	Three different structured CNN models+inception	Good performance and robust generalization.	A sufficient number of the same types of soil samples with similar physical structures were required; the experimental results of the algorithm were heavily influenced by preprocessing such as feature selection.
Oct. 2020, [133]	Coal	Improved coyote optimization algorithm (I-COA) +local receptive field-based–extreme learning machine (LRF–ELM)	Improved the economy, speed, and accuracy while more effectively extending the spectral properties of coal.	Long training time.
Dec. 2020, [134]	Soil	A joint convolutional neural network and recurrent neural network architecture (CCNVR)	A significant improvement in prediction accuracy and a better ability to migrate.	With fewer training samples, the model’s robustness and accuracy will significantly decrease.
Jun. 2021, [135]	Salt content in saline-alkali soil (SAS)	Convolutional neural network–gravitational reservoir-computing–extreme learning machine (CNN-GRC-ELM).	A fast, low-cost, and accurate method.	Does not compare to other state-of-the art deep learning models; the experimental sample data were insufficient.
Jun. 2022, [136]	Polyethylene	β-variational autoencoder (β-VAE)	Improved ability to analyze spectroscopic data from complex heterogeneous systems.	More algorithms needed to be compared than with the PCA algorithm.
Oct. 2020, [137]	Water pollution	An improved convolutional neural network (CNN)+decision tree	Improved NIR prediction accuracy; rapid determination.	Only preliminary experimental results; the decision tree’s parameters had an impact on the model’s performance.
Jan. 2020, [138]	Cereal	Stacked sparse autoencoder (SSAE)+affine transformation (AT)+extreme learning machine (ELM)	A quick, effective, and economical method for analyzing cereal characteristics with good prediction results.	The test samples were insufficient in terms of quantity and variety.
May. 2020, [139]	Cells	Mie extinction–extended multiplicative signal correction (ME-EMSC)	In terms of the speed, robustness, and noise levels, the DSAE performed better than Mie extinction–extended multiplicative signal correction (ME-EMSC).	In the experimental preliminary results, a sizable number of additional experimental samples were required.
Nov. 2020, [140]	Soil	CNN	When the number of calibration samples exceeded 2000, the CNN was more accurate than the machine learning models.	Larger datasets should be explored to test the generalization of the accuracy vs. sample size and explore whether the deep learning CNN model ever reaches a plateau in accuracy.
Nov. 2020, [141]	Physical distortions	Extended multiplicative signal augmentation (EMSA)+SpectraVGG	The convergence occurred much more quickly and the results were better.	The final model results were strongly influenced by the methods used for data augmentation and signal preprocessing.

The encoder output of a single-layer AE is given by

(3) $$h=f(xW_{1}+b_{1})$$

where f() is a nonlinear activation function such as sigmoid and $W_{1}$ and $b_{1}$ are the weights and bias of the encoder, respectively. The decoder output of a single-layer AE is given by

(4) $$z=f(h{W_{2}}^{T}+b_{2})$$

where *z* is the reconstructed input *x* and $W_{2}$ and $b_{2}$ are the weight and bias of the decoder, respectively. The following objective function is minimized by the AE and SAE:

(5) $$J_{r}=E_{x}\left[||x-{z||}_{2}^{2}\right]$$

where $E_{x}$ is the expected value. Finally, the SAE decoder layer is removed and, depending on the task, a classification or regression layer is added. Other SAE variants, such as the stacked contractive autoencoder (SCAE) [142], learn a low-dimensional manifold that is invariant to small changes and is given by

(6) $$J_{c}=J_{r}+\sum_{i}\sum_{j}{\left(\frac{\delta (h_{i})}{\delta x_{j}}\right)}^{2}.$$

The stacked denoising autoencoder (SDAE) [143] learns a low-dimensional manifold that is noise-invariant and given by

(7) $$J_{d}=E_{x}\left[\left|\right|\tilde{x}-z{\left|\right|}_{2}^{2}\right]$$

where $\tilde{x}$ is randomly corrupted input data.

A low-dimensional manifold is learned by the AE, which is advantageous for a subsequent task such as classification or regression.

A VAE is a generative model that generates samples by modeling the joint distribution P(x,z). The joint distribution is calculated by modeling the conditional distribution P(x|z) and multiplying by P(z) as P(x,z)=P(x|z)P(z). As a result, the VAE encoder maps the input data to a low-dimensional manifold with Gaussian distribution since the joint distribution P(x,z) can be calculated if the distribution of the low-dimensional manifold is known. Therefore, the encoder can be removed from the VAE and a random Gaussian distribution value can be generated and passed to the decoder to generate a sample. The VAE optimization is given by

(8) $$J_{svae}=J_{sae}+KL\left(q\left(z|x\right)||N\left(0,1\right)\right)$$

where N(0,1) is the normal distribution with a mean of zero and a variance of one and q(z|x) is the approximated output distribution of the encoder.

### 7.2. Convolutional Neural Network (CNN)

**Figure 4:**
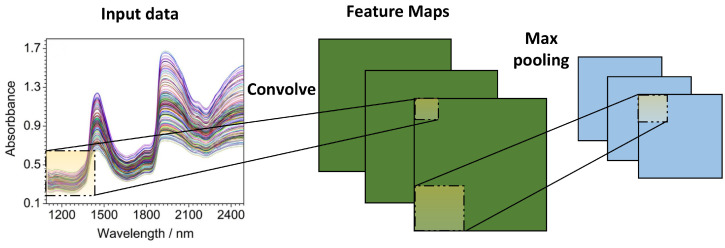
Illustration of a convolutional and pooling layer used in CNN.

A CNN learns local features such as NIR data peaks by employing a series of convolutional and pooling layers, followed by a fully connected layer for classification or regression. Convolutional layer weights are referred to as kernels and each kernel connects only to a portion of the input data referred to as the local receptive field (LRF). To process all of the input data, the kernels are moved from one end to the other, covering all of them with overlap. The kernel size, kernel movement or stride, and number of kernels are the hyperparameters given by the user. The convolutional layer outputs a feature map for each kernel by calculating the dot product of each LRF and kernel. Each kernel connects only to an LRF, allowing the kernel to acquire local features. There are two different outputs from a convolutional layer: (1) same; and (2) valid. In order to give the feature map after the convolutional operation the same dimension as the input data, the same convolutional layer increases the dimension of the input data by padding zeros. The input data are not padded by the valid convolutional layer and the feature map has a lower dimension than the input data. The 2D convolutional operation is given by

(9) $$h{\left(k\right)}_{1}=\sum_{i}\sum_{j}W_{1}\left(k\right)\cdot x\left(i,j\right)$$

where $W_{1}\left(k\right)$ is the *k*-th kernel and $h{\left(k\right)}_{1}$ is the *k* feature map of the convolutional operation. The feature map is downsampled by the addition of a pooling layer after a convolutional layer. To learn the high-level features, the pooling layer combines the low-level features. The pooling layer consists of a kernel with fixed values that moves from one end to the other covering all previous layers’ outputs and uses a maximum or mean operation to calculate the output instead of the dot product. The 2D max-pooling operation is given by

(10) $$h{\left(k\right)}_{2}=maxi_{(i,j)\in R}h{\left(k\right)}_{1}\left(i,j\right)$$

where R is the region of max pooling that is used. The convolutional and pooling operations are illustrated in Figure 4.

When designing a CNN, the main research direction has been to improve the generalization capability of the CNN by increasing the layers and resolving the problems that are encountered when increasing the layers. AlexNet was the first CNN applied to image classification tasks and was shown to be capable of using the graphical processing unit (GPU) to reduce the training time [144]. VGGNet [145] further expanded AlexNet by increasing the number of layers from 8 to 19 and reducing the kernel sizes from 11 to 3, which improved the model’s generalization ability. One of the main issues with increasing the number of CNN layers beyond 19 is that the initial layers are not updated as the gradients become smaller at each layer or explode as a result of the large error of the deep CNN network. ResNet [146] was the first network to use 152 layers and solved the vanishing and exploding gradient problem with skip connections between a block of layers. However, some layers did not acquire any useful capabilities. To avoid this issue, Wide ResNet [147] reduced the number of layers to 40 while increasing the number of feature maps of each layer.

The CNN architecture uses various types of layers to learn a model that maps the input data to the output data and captures important features. As a result, different CNN architectures are employed for different types of data. For example, for speech-to-text conversion, a CNN architecture with convolutional and recurrent layers was used [148] because the recurrent layers can capture long-term temporal information. DeepSpectra, a CNN architecture with three convolutional layers and an inception module, was also introduced for NIR data [149]. The inception module consisted of parallel convolutional layers with different kernel sizes. The inception module trained sparse filters by distributing the features among parallel convolutional layers to improve generalization capacity. NIR data features were sparse because only a few features described the composition of the sample and were captured by the convolutional layers with the inception module in DeepSpectra.

### 7.3. Recurrent Neural Networks (RNNs) and Attention

An RNN is used for time-series data in natural language processing (NLP), speech-emotion recognition (SER), speech-to-text (STT), and other processes [150]. Since wavelengths are measured at regular intervals, the wavelength axis is regarded as the time-series axis in NIR data.

There are many types of RNNs such as simple RNNs, long short-term memory (LSTM) [151], and gated recurrent units (GRUs) [152]. An RNN computes the hidden layer based on the previous time step, and the output of a simple RNN’s hidden layer is given by

(11) $$h\left(t\right)=f(W_{1}x\left(t\right)+W_{2}h\left(t-1\right)+b_{1})$$

where $W_{1}$ is the weight input data, $W_{2}$ is the weight of the previous hidden layer’s output h(t−1), and $b_{1}$ is the bias. The vanishing and exploding gradient problem affects simple RNNs with long time steps. LSTM solves the vanishing gradient problem by using memory to remember the gradients of previous time steps. A GRU is a streamlined version of LSTM with lower computational complexity. GRUs have been shown to perform similarly to LSTM on datasets with a limited number of data samples, but LSTM outperforms GRUs on datasets with a large number of samples.

RNNs output a series of hidden states at each time step when used for classification or regression, with the most recent state or statistics from the hidden states, such as the mean or standard deviation, used as inputs. However, these methods do not always choose the most appropriate hidden state. In contrast, an attention mechanism can be used to select the best-hidden state for an RNN for classification or regression.

**Figure 5:**
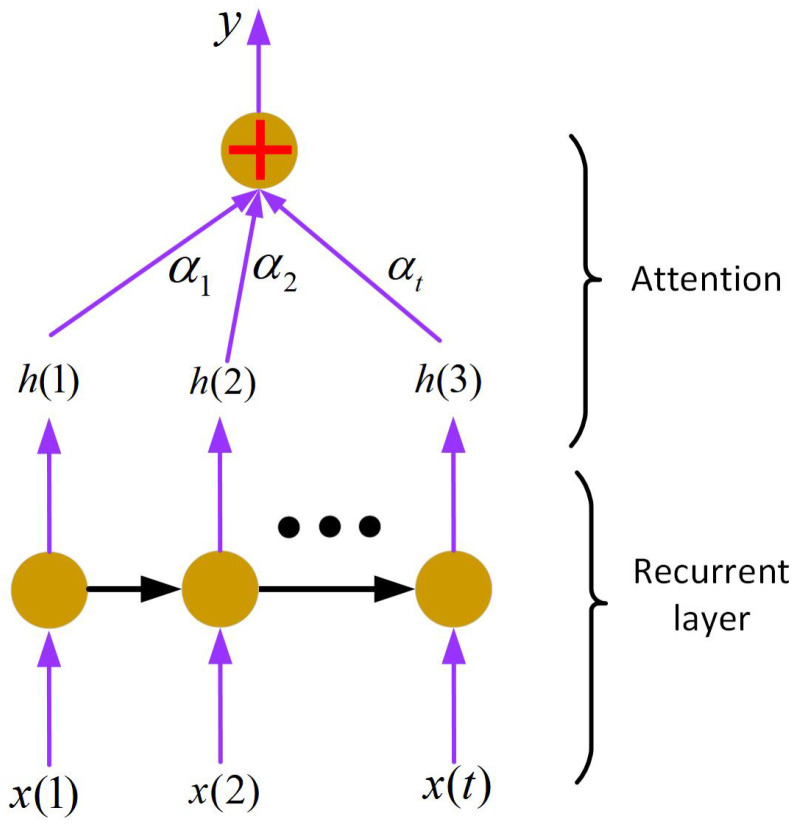
Recurrent layer with an attention mechanism.

In language translation, attention is first introduced to identify key words. Figure 5 shows a recurrent layer, which is followed by an attention mechanism. Attention achieves this by using weights to quantify the significance of each RNN’s output, which is given by

(12) $$s=\sum_{t}^{T}\alpha \left(t\right)h\left(t\right)$$

where α(t) is a weight learned by attention to measure the importance of the RNN’s hidden state and is calculated as

(13) α(t)=softmax(v(t))

where $W_{3}$ is a learnable weight and v(t) is an SLFN output, which learns the significance of the RNN’s hidden state and is given by

(14) $$v\left(t\right)=W_{4}tanh\left(W_{3}h\left(t\right)+b_{2}\right)$$

### 7.4. Deep Extreme Learning Machine Architectures

ELM was first introduced as SLFN and has demonstrated good performance for data with a limited number of training samples such as NIR data [153]. Due to the fact that other deep architectures require a large number of training samples, ELM was extended to deep architectures in order to improve the generalization capability for data with few training samples.

Multilayer–extreme learning machine (ML-ELM) is developed by stacking extreme learning machine–autoencoder (ELM-AE) output weights in order to learn a low-dimensional manifold with a limited number of training samples. In contrast to SAE, ML-ELM employs a more straightforward training methodology, where the classification or regression layer is learned after a single step of greedy layer-wise training. As a result, ML-ELM does not require any fine-tuning, which shortens the training period. Furthermore, the ELM-AE’s input weights are chosen at random and are not tuned, whereas the output weights are calculated using a closed-form solution. The ELM-AE’s hidden layer output is calculated as follows:

(15) $$h=f(xW_{1}+b_{1})$$

where f() is a nonlinear activation function such as sigmoid and $W_{1}$ and $b_{1}$ are the untuned random weights and biases. The ELM-AE’s output is as follows:

(16) $$z=hW_{2}^{T}$$

where *z* is the ELM-AE’s output and $W_{2}$ is the output weight of the ELM-AE calculated using a closed-form solution as follows:

(17) $$W_{2}^{T}={\left(H^{T}H+1/C\right)}^{-1}H^{T}X$$

where *C* is a hyperparameter given by the user. In the case of a one-layer ML-ELM, the first hidden layer of the ML-ELM is calculated as follows:

(18) $$h_{1}=f\left(xW_{2}\right).$$

The ELM-AE calculates and stacks the subsequent layers of ML-ELM weights, as shown in Equations (17) and (18), respectively.

An LRF-ELM is a CNN architecture with one or more convolutional layers followed by a max-pooling layer and a classification or regression layer. In contrast to conventional convolutional layers, which update their kernel weights using back-propagation, the LRF-ELM’s kernel weights are orthogonally random and not tuned. Singular value decomposition (SVD) is applied to the random kernel weights to produce orthogonal random kernel weights. Features that are randomly scaled and rotated are contained in random kernel weights, whereas the features in the orthogonal random weights are simply rotated at random. Randomly scaled features are rendered useless and only randomly rotated features are useful, as the pooling layer introduces scale invariance.

### 7.5. Generative Adversarial Networks (GAN)

**Figure 6:**
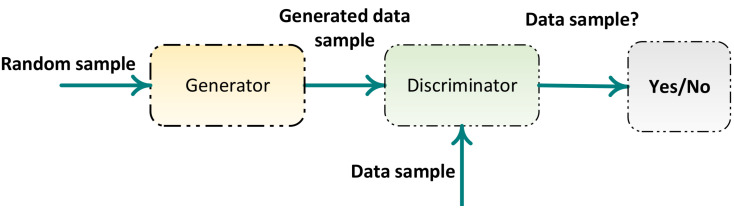
The structure of a GAN network.

Due to the limited number of training samples available for NIR data, GAN is utilized to generate new training samples [154]. GAN is a generative model composed of a generator network that generates data from a random vector sampled from a Gaussian distribution and a discriminator that attempts to determine whether the generated data resemble the actual data, as depicted in Figure 6. Therefore, the goal is to maximize discriminator loss while minimizing generator loss. Fully connected neural network, CNN, or RNN architectures, which are selected based on the data type, could be used as the generator and discriminator. A CNN+RNN architecture for the generator and discriminator would be appropriate for NIR data because it can capture local features, as well as long-term relationships between the wavelengths. Zheng et al. proposed a modified bidirectional generative adversarial network (BI-GAN) method for classifying the near-infrared spectra of drugs, given the dilemma of insufficient samples within a class and sample imbalance between classes. The results demonstrate that CNN-based modeling outperformed the stacked autoencoder, which had the highest error rates when estimating soil characteristics [155]. Yang et al. proposed machine learning algorithms combined with NIR spectroscopy to accurately distinguish cumin and fennel from three different regions, Turpan, Yumen, and Dezhou, with all model parameters remaining unchanged [156]. The GAN optimization is as follows:

(19) $$minS_{G}maxS_{D}V\left(D,G\right)=E_{x}\left[log(D(x))]\right]+E_{z}\left[log(1-D(G(z))\right]$$

where D() is the discriminator, G() is the generator, and *z* is the random vector sampled from a Gaussian distribution. A GAN classifies generated and real data using a two-step learning process, where the first step only updates the discriminator weights. In the second step, the generator weights are modified to generate data in an attempt to deceive the discriminator into believing it is real data. The majority of architectures described in the literature use ML algorithms to map the NIR absorption values to the desired output.

In contrast to VAE, which uses the mean square error (MSE) to measure reconstruction, a GAN uses a discriminator model to measure reconstruction. The MSE is not an appropriate metric for measuring reconstruction because it does not account for the significance of the features. As a result, GAN reconstructions have been perceived by users to be more realistic than VAE reconstructions.

### 7.6. Discussion

Although most traditional machine learning methods are used for NIR data from liquid or gas samples, deep architectures are used for NIR data from solid samples. Deep architectures can reduce the variance introduced by solid samples, which have variable path lengths due to scattering. Deep architectures are used in NIR to determine the quality of medicines [125,126,127,157], meats, vegetables and fruits [128,129,130,131,132], the contents of soil and minerals [13,133,134,135,158], cracks in water pipes [136], the manufacturing date of paper [159], and water pollution [137], as well as in the cursory evaluation of malady [160]. Experiments conducted on four distinct datasets containing solid samples demonstrated that the number of training samples influences the generalization ability of deep learning algorithms [140,141].

NIR data have a limited number of training samples due to the difficulty of obtaining labeled data. Generating data using a GAN is one method for increasing the number of training samples [154]. However, the generalization ability of the machine learning algorithm can be influenced by the quality of the generated data. Therefore, As a result, a labeler should evaluate the generated data to determine whether they are of sufficient quality for training.

Deep architectures such as SAE and VAE are used as a nonlinear feature reduction approach as opposed to the PCA feature reduction approach commonly used by traditional machine learning methods for NIR data. The feature reduction approach is nonlinear in SAE and VAE, there is no mapping with the reduced features, and non-reduced NIR data features cannot be interpreted.

Deep architectures are also used for preprocessing, which aims to eliminate scattering-related variance. To accomplish this, a convolutional encoder–decoder architecture that outperformed other preprocessing functions was introduced [139].

## 8. Conclusions

Machine learning algorithms for NIR spectroscopy research have been reviewed in this paper. The processing of NIR spectroscopy using ML algorithms is a widely used, rapid, and non-invasive method for determining the composition of the target sample. The majority of architectures reported in the literature utilized ML algorithms to map NIR absorption values to the desired output. There are two types of network architectures for ML algorithms: traditional machine learning methods and deep network architectures. Deep network architectures have many hidden layers, whereas traditional machine learning methods only have a few or no layers. Deep network architectures use raw features, whereas traditional machine learning methods call for the expert engineering of suitable features. In contrast to deep network architectures, the performance of traditional machine learning methods depends on the features that were engineered. As a result, the prevalence of deep network architectures is increasing, as experts are no longer required to engineer features. The preprocessing of noisy data to remove additive and multiplicative bias noise is required before ML algorithms can perform as intended since the space available for batteries and the powerful processing of components in portable NIR devices is limited. Another significant aspect is feature selection, which identifies the specific wavelengths that describe the composition of a sample and reduces the number of features, reducing the computational complexity of machine learning models. In summary, the integration of a variety of machine learning algorithms in an efficient and lightweight manner is the goal of future research and development efforts.

## Figures and Tables

**Table 1:** Comparison of different NIR instruments.

Instrument	Cost	Speed	Signal to Ratio
LED	very low	moderate	moderate
AOTF	moderate	very fast	low
Dispersive	low	slow	high
Fourier	high	fast	very low

## Data Availability

Not applicable.
